# Age at menarche and childhood body mass index as predictors of cardio-metabolic risk in young adulthood: A prospective cohort study

**DOI:** 10.1371/journal.pone.0209355

**Published:** 2018-12-21

**Authors:** Chi Le-Ha, Lawrence J. Beilin, Sally Burrows, Rae-Chi Huang, Martha Hickey, Trevor A. Mori, Roger J. Hart

**Affiliations:** 1 Medical School, Royal Perth Hospital Unit, University of Western Australia, Perth, Western Australia, Australia; 2 Telethon Kids Institute, University of Western Australia, Subiaco, Western Australia, Australia; 3 Department of Obstetrics & Gynaecology, University of Melbourne, Royal Women’s Hospital, Parkville, Victoria, Australia; 4 Division of Obstetrics and Gynaecology, University of Western Australia, King Edward Memorial Hospital, Perth, Western Australia, Australia; Medizinische Universitat Innsbruck, AUSTRIA

## Abstract

**Objective:**

This study aimed to examine the association between age at menarche and a range of cardiovascular disease (CVD) risk factors at 17 and 20 years of age, and whether this was influenced by childhood body mass index (BMI).

**Methods:**

Of the 1413 girls born in the Western Australian Pregnancy Cohort (Raine) Study, 846 had age at menarche recorded. Subsequently 557 underwent metabolic assessment at 17 years and 541 at 20 years. Associations between age at menarche and cardiovascular risk factors, and being in a high-risk metabolic cluster at 17 and 20 years, or having the metabolic syndrome at 20 years, were investigated by linear mixed effects and logistic regressions, respectively.

**Results:**

Each year later of onset of menarche was associated with a 0.75 kg/m^2^ reduction in BMI (coefficient -0.75 [95%CI -1.06, -0.44]), and an approximate 30% reduction in the odds of being in the high-risk metabolic cluster at 17 years (OR = 0.73 [95%CI 0.57, 0.94]) and 20 years of age (OR = 0.68 [95%CI 0.52, 0.87]), and a 40% reduction in the odds of having the metabolic syndrome at 20 years (OR = 0.60 [95% CI 0.41, 0.88]). These data show earlier age at menarche was associated with increased BMI and odds of being in the high-risk metabolic cluster at 17 and 20 years, and increased odds of having the metabolic syndrome at 20 years. However, these associations were no longer statistically significant after adjustment for BMI at age 8 years. Current smoking, alcohol consumption, physical activity, socio-economic status, or hormonal contraceptives use did not affect these associations.

**Conclusions:**

Earlier age at menarche may be indicative of a higher risk profile for CVD in young adulthood. Our findings suggest that targeted interventions to reduce BMI in girls who experience menarche at younger age may reduce CVD risk in the future.

## Introduction

Cardiovascular disease (CVD) is the leading cause of mortality in women in Western countries [1], and it has been suggested that the origins of CVD may initiate at an early age. Hence identification and intervention for these early life modifiable factors to prevent CVD in later life is essential [2]. The American Heart Association state [3]: “The primary focus is on adult cardiovascular health and disease prevention, but critical to achievement of this goal is maintenance of ideal cardiovascular health from birth through childhood to young adulthood and beyond.” Studies have shown that atherosclerotic disease is present at an early age. For example, over 70% of American soldiers killed in the Korean war, with a mean age of 22 years, had documented presence of atherosclerosis at post-mortem examination [4]. Hence identification of childhood cardiovascular risk factors that may predict adult cardiometabolic risk is essential.

Earlier onset of puberty and age at menarche have been linked to a broad range of adult chronic diseases [5], including cardio-metabolic health in adulthood [6, 7]. Earlier age at menarche has been associated with increased levels of risk factors for CVD including obesity and hypertension [8], left ventricular dysfunction [9], type 2 diabetes [10], and the metabolic syndrome [11] in adulthood. Moreover, there is evidence for a relation between menarche and morbidity from CVD, with results from meta-analysis suggesting an association between early menarche and higher CVD related risk [12]. However, most published studies have relied on retrospective recall of age at menarche, sometimes over many decades, which is not considered reliable [13], and have not adjusted for key drivers of menarche such as childhood BMI or lifestyle factors such as smoking which likely modify this association [12]. Despite extensive published studies of the timing of age at menarche and its relationship with greater risk of CVD [7], there is little understanding of the mechanisms involved. Greater childhood BMI is a predictor of earlier age at menarche [14], and is associated with increased CVD risk if it persists into adulthood. However it is not clear whether earlier age at menarche is an independent risk factor for CVD in adulthood or whether greater childhood BMI is a major confounder in the relationship between age at menarche and increased CVD risk later in life [15]. These issues are of increasing importance, given the global increase in childhood overweight and obesity.

We have previously demonstrated that in girls from the Western Australian Pregnancy Cohort (Raine) Study, being born with a birthweight below expected by gestation at delivery with adjustment for mother’s height, age and parity, and subsequently having a body mass index (BMI) above average at 8 years of age, was a significant predictor of early age at menarche [16]. Consequently within this same contemporary cohort of girls, we aimed to investigate the association between age at menarche and a range of CVD risk factors at 17 and 20 years of age, and whether this was influenced by childhood BMI.

## Methods

### Study population

**Fig 1:**
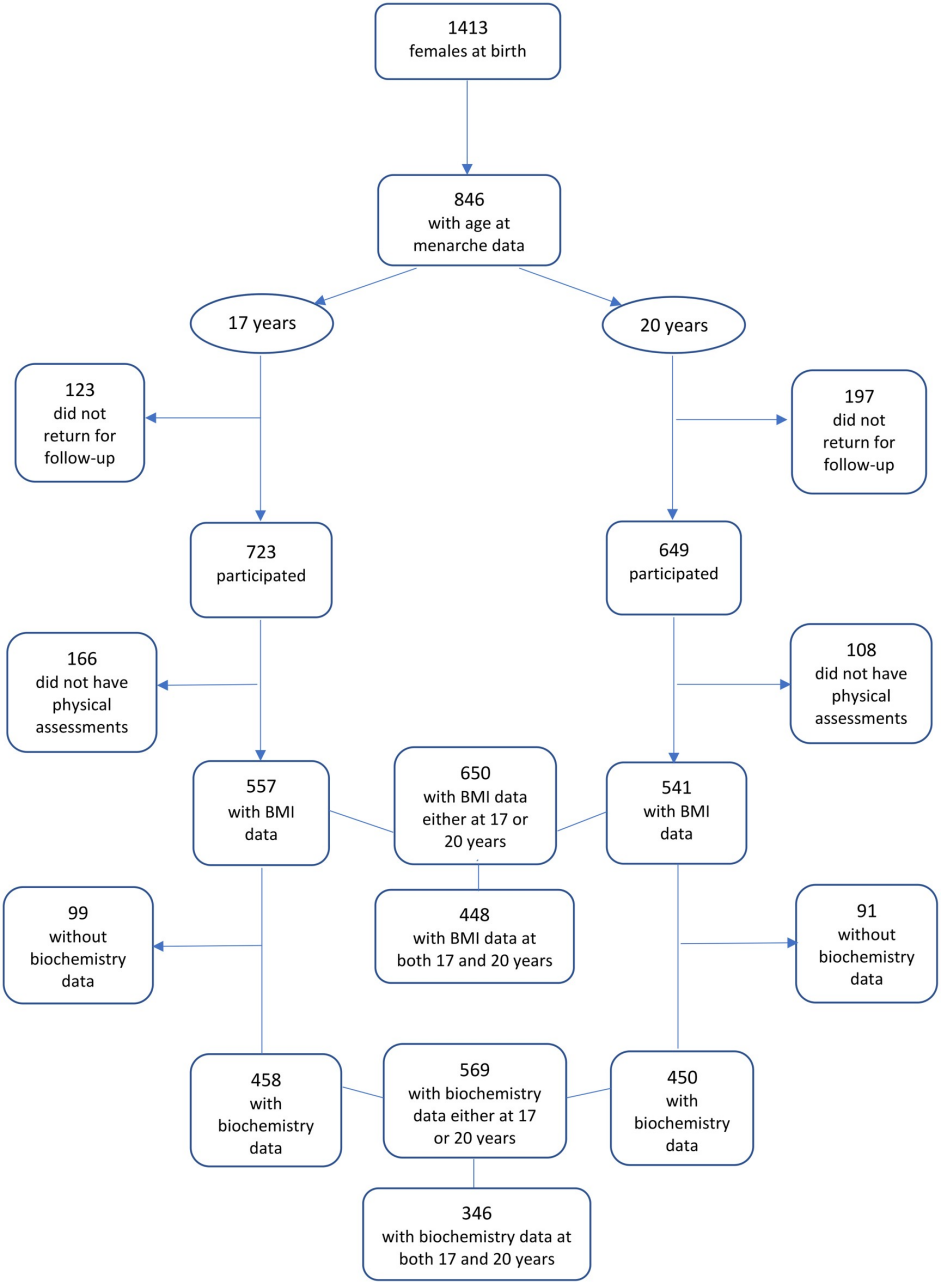
Flow diagram of females attending the Raine Study 17-year and 20-year reviews.

The Western Australian Pregnancy Cohort (Raine) Study, is an on-going longitudinal population-based cohort study in which 2868 live births from 2900 women recruited from King Edward Memorial Hospital and nearby clinics in Perth, Western Australia, were enrolled at 18 weeks of pregnancy between 1989 and 1991. The study population was comprised of 85% Caucasian women. Details of the study are published elsewhere [17]. The study was approved by the Human Ethics Committees of King Edward Memorial Hospital and Princess Margaret Hospital for Children in Perth. Written informed consent was obtained from the mother/primary care giver and the participants at 17 and 20 years of age. The current analysis was based on data from girls participating in assessments at 8, 10, 14, 17 and 20 years of age, and information obtained from the mothers during pregnancy. Fig 1 provides a flow diagram of the study samples. As in most longitudinal studies, loss to follow-up from the original population is inevitable. However, the Raine Study participants remain representative of the Western Australian population, and participants and non-participants at each cohort review remain constant across a number of socio-anthropological and clinical characteristics [18].

### Age at menarche

Information on age at menarche calculated to the nearest month was obtained using a purpose-designed questionnaire [19] at 8, 10, 14 and 17 years of age. Mothers or caregivers were asked whether their daughter had experienced menarche since the last study follow-up examination, and to report the exact date of the onset of the first and subsequent 3 menstrual periods. Menarche was defined as the date of the onset of the first menstrual period [20].

### Anthropometric and clinical data

At 17 and 20 years, fasting venous blood samples were obtained for the assessment of glucose, insulin, total cholesterol, triglycerides, high-density lipoprotein cholesterol (HDL-C), and low-density lipoprotein cholesterol (LDL-C). Homeostasis model of assessment for insulin resistance (HOMA-IR), an estimate of insulin resistance was calculated as insulin (mU/L) x glucose (mmol/L) / 22.5. After resting for 5 minutes, supine systolic blood pressure (SBP) and diastolic blood pressure (DBP) were recorded using an oscillometric sphygmomanometer (Dinamap Pro Care 100; Soma Technology, Bloomfield, Connecticut, USA), and the last 5 of 6 readings were averaged. Wearing minimal clothing and without shoes, participants were weighted with a Wedderburn Chair Scale (nearest 100 g), and height measured with a Holtain Stadiometer (nearest 0.1 cm). The metabolic syndrome was defined according to the criteria of the International Diabetes Federation [21]: waist circumference ⩾ 90th percentile and at least two of the following factors present: (a) triglycerides ⩾ 1.7 mM; (b) HDL-C < 1.03 mM; (c) SBP ≥ 130 mmHg or DBP ≥ 85 mmHg; and (d) fasting plasma glucose ≥ 5.6 mM.

Hormonal contraceptive (HC) use information was obtained from the question, ‘In the last 6 months, have you taken any prescription medication(s) e.g. the Pill?’ (if yes, ‘which medication(s), and are you still taking it?’). At year 20, HC use included any forms of HC including the progestogen-releasing intrauterine device and subdermal implants.

Maternal pre-pregnancy weight was obtained from self-report, and height was measured at 18 weeks of gestation.

### Socio-behavioural data

Socio-behavioural features at 17 and 20 years were assessed via a computer-based questionnaire. An alcohol drinker was defined as consuming alcohol at any level in a day during the last 7 days. The number of cigarettes consumed each day in the last 7 days was recorded, from the questions ‘Have you ever smoked cigarettes in the past 12 months?” and “Have you smoked cigarettes in the past 4 weeks?’ Maternal smoking in pregnancy was self-reported by the mother from the question ‘Do you smoke now?’ with the number of cigarettes consumed. Physical activity at year 17 was assessed from the question, ‘How many hours do you usually exercise in your free time in a week, so much that you get out of breath or sweat?’, from which a dichotomous variable with the cut-point at ≥ 4 hours per week was created. Socioeconomic status was assessed from annual family income (Australian dollars) at 17 years.

In our investigations, *a priori* variables included maternal smoking in pregnancy, maternal pre-pregnancy BMI, birth weight, BMI at 8 years, physical activity and family income at 17 years, and cigarette smoking, alcohol consumption, and HC use at 17 and 20 years. These factors have been identified as being potentially associated with cardiovascular outcomes.

### Statistical analysis

Age at menarche was analysed as a continuous variable; however, for the purpose of describing the sample characteristics, a 3-level categorical variable for age at menarche (≤ 11y, 12-13y, and ≥ 14y) was constructed. CVD risk factors were all analysed as continuous variables. A binary metabolic cluster outcome identifying high and low metabolic risk (separately for 17 yr and 20 yr) was also investigated. This cluster variable was derived from triglycerides, BMI, HOMA-IR, and SBP, using a two-step cluster analysis [22], involving a scalable cluster algorithm. The linearity of the relationship between age at menarche and a continuous outcome was assessed with multivariable spline modelling.

Hierarchical linear and logistic mixed effects regression models were employed to assess the differences between 17 and 20 years for continuous and categorical CVD variables (with the xtmixed and xtmelogit procedures in Stata, respectively). These models utilise maximum likelihood estimation (MLE) which retains all available data including participants with only a single follow up assessment in the analysis. This is known to result in unbiased estimates if missing data are missing at random. Linear mixed models with MLE were also employed to examine the relation between age at menarche and the development of CVD risk factors from years 17 to 20. Time was treated as a 2-level factor variable. Hierarchical models were required to adjust for potential correlation between a small number of siblings present in the sample. Because insulin data were left censored, Tobit regression (the xttobit procedure) was used for analysis. The xttobit command does not allow an adjustment for correlated siblings data, however bootstrapping was employed to obtain robust standard errors.

Models for each CVD risk factor initially included age at menarche and time, to account for the change of the risk factor between 17 and 20 years of age. In models with significant associations with age at menarche, BMI at age 8 years was then included to examine the effect of childhood adiposity on the relationship. The remaining *a priori* covariates were then added and a manual backward stepwise elimination process employed, in which non-significant covariates in the model were removed, with specific focus on any change in the coefficient for age at menarche that signified confounding, and p-values at each step. Retained in the final multivariable models were all factors still significantly associated with the outcome or influential in the model. Further, to investigate the influence of *a priori* covariates on this relationship, the interaction between age at menarche and each covariate was tested. The Holm’s step-down procedure was used to identify a significant association after adjusting for multiple comparisons.

Given the sample specific derivation of the metabolic cluster variable at each time point, cross-sectional logistic regression analyses were performed for each year, along with the metabolic syndrome at year 20, to investigate the associations with age at menarche. A per family cluster variance adjustment was incorporated to account for potential correlation between siblings. The model building approach used for continuous outcomes was also adopted for the metabolic outcomes. Due to the small number of participants having the metabolic syndrome, the final model for this variable was bootstrapped to obtain p values that were robust to overfitting.

All analyses were performed using Stata version 13 (StataCorp, College Station, Texas, USA). Results are interpreted with statistical significance set at p < 0.05.

## Results

The phenotypic, biochemical and socio-behavioural characteristics of 650 girls, who had complete data on age at menarche and BMI either at 17 or 20 years (n = 557 and n = 541, respectively) are presented in Table 1. Among these participants, 448 had data at both 17 and 20 years. The average age at menarche was 12.7 years (95% CI 12.6, 12.8). Participants’ BMI, SBP, DBP, cholesterol, LDL-C and glucose levels increased over the 17 to 20 year period. Insulin levels and HOMA-IR were significantly higher at age 17 years compare to age 20 years. These data corroborate with recent observations of an increase in insulin resistance in adolescents during the pubertal period [23]. At 20 years of age, 60% were HC users, 68% consumed alcohol, and 12% smoked cigarettes. The prevalence of smoking declined significantly over the period of 17 to 20 years. Approximately 22% of the participants’ mothers smoked in pregnancy. Descriptive characteristics of the study participants at years 17 and 20 according to a 3-level age at menarche (≤ 11y, 12-13y, and ≥ 14y) are demonstrated in Table 2.

**Table 1:** Characteristics of the study participants (N = 650).

	Early life to menarche	Year 17 (n = 557)	Year 20 (n = 541)	P value
Maternal smoking in pregnancy %	22.5			
Maternal pre-pregnancy BMI, kg/m^2^	22.4 (4.3)			
Age at menarche, yr	12.7 (1.1)			
BMI at year 8, kg/m^2^	16.8 (2.5)			
BMI in early adulthood, kg/m^2^		23.1 (4.5)	24.3 (5.4)	<0.001
SBP, mmHg		108.8 (9)	111.1 (9.9)	<0.001
DBP, mmHg		59.4 (6.4)	65.4 (7.2)	<0.001
Total cholesterol, mmol/L		4.3 (0.7)	4.5 (0.7)	<0.001
Triglycerides, mmol/L		1.0 (0.5)	1.1 (0.5)	0.228
HDL-C, mmol/L		1.4 (0.3)	1.5 (0.3)	<0.001
LDL-C, mmol/L		2.4 (0.6)	2.6 (0.6)	<0.001
Glucose, mmol/L		4.6 (0.4)	4.9 (0.7)	<0.001
Insulin, mU/L †		8.0 (5.4, 11.4)	2.8 (1.9, 6.0)	<0.001
HOMA-IR †		1.6 (1.1, 2.4)	0.6 (0.4, 1.3)	<0.001
Smoker *, n %		124 (22.3)	62 (12)	<0.001
Alcohol drinker ǂ, n %		281 (50.5)	445 (68.4)	<0.001
HC user, n %		175 (30.3)	335 (60.3)	<0.001
High-risk metabolic cluster, n %		87 (19.1)	96 (21.5)	N/A¶
Metabolic Syndrome, n%			24 (4.3)	
High physical activity §, n %		105 (21)		
Annual family income at year 17, n %				
≤A$35 000		69 (12.9)		
A$35 001 to ≤ A$78 000		177 (33.1)		
>A$78 000		289 (54)		

Data are expressed as mean (standard deviation), median (Q1, Q3) †, or n (percentage).

Abbreviations: BMI, body mass index; SBP, systolic blood pressure; DBP, diastolic blood pressure; HDL-C, high-density lipoprotein cholesterol; LDL-C, low-density lipoprotein cholesterol; HOMA-IR, homeostasis model of assessment for insulin resistance; HC, hormonal contraceptives.

* Smoking ≥ 1 cigarette in a week.

^ǂ^ Consuming alcohol at any level over the last 7 days.

^§^ Having ≥ 4 hr of exercise in free time per week.

^¶^ Different criteria were used for years 17 and 20, respectively.

**Table 2:** CVD risk factors of the study participants by categories of age at menarche (N = 650).

	Year 17 (n = 557)			Year 20 (n = 541)		
*Categories of age at menarche*	*≤ 11y* *(n = 124)*	*12-13y* *(n = 358)*	*≥ 14y* *(n = 75)*	*≤ 11y* *(n = 118)*	*12-13y* *(n = 351)*	*≥ 14y* *(n = 72)*
BMI at year 8, kg/m^2^	17.7 (3.2)	16.6 (2.2)	16.2 (2.4)	17.4 (2.9)	16.6 (2.2)	15.9 (1.9)
BMI in early adulthood, kg/m^2^	24.2 (5.3)	22.8 (4)	22.7 (5.3)	25.4 (5.9)	24.1 (5)	23.8 (5.9)
Waist circumference, cm †	76.1 (71, 8)	75 (69.6, 82.3)	75.3 (70.3, 82.9)	75.6 (69.8, 82.8)	73.9 (68, 81.8)	74.4 (68.7, 81.5)
SBP, mmHg	109.1 (8.9)	108.9 (8.9)	107.9 (9.4)	112.1 (10.3)	110.9 (10.1)	110.3 (8.8)
DBP, mmHg	59.7 (6.3)	59.7 (6.5)	57.8 (6.1)	65.6 (6.6)	65.4 (7.4)	64.8 (6.7)
Total cholesterol, mmol/L	4.4 (0.7)	4.3 (0.7)	4.23 (0.7)	4.6 (0.7)	4.5 (0.8)	4.6 (0.8)
Triglycerides, mmol/L	1.0 (0.4)	1.0 (0.5)	1.0 (0.5)	1.0 (0.5)	1.1 (0.4)	1.1 (0.5)
HDL-C, mmol/L	1.4 (0.3)	1.4 (0.3)	1.4 (0.3)	1.5 (0.3)	1.5 (0.3)	1.5 (0.3)
LDL-C, mmol/L	2.5 (0.6)	2.4 (0.7)	2.4 (0.6)	2.6 (0.6)	2.5 (0.6)	2.6 (0.6)
Glucose, mmol/L	4.6 (0.4)	4.6 (0.4)	4.7 (0.4)	4.9 (0.4)	4.9 (0.8)	4.8 (0.3)
Insulin, mU/L †	8.3 (5.3, 12.3)	7.9 (5.42, 10.9)	8.3 (5.9, 11.5)	3.2 (1.9, 6.0)	2.9 (1.9, 6.0)	2.6 (1.9, 6.1)
HOMA-IR †	1.7 (1.0, 2.5)	1.6 (1.1, 2.3)	1.6 (1.3, 2.5)	0.6 (0.4, 1.3)	0.6 (0.4, 1.4)	0.6 (0.4, 1.3)
High-risk metabolic cluster, n (%)	27 (26.7)	46 (15.9)	14 (21.2)	30 (30.6)	55 (19.2)	11 (17.7)
Metabolic Syndrome, n (%)				9 (7.4)	12 (3.3)	3 (4.2)
Smoker *, n (%)	29 (23.5)	82 (22.8)	13 (17.3)	12 (11)	42 (12.5)	8 (11.4)
Alcohol drinker ǂ, n (%)	58 (46.8)	190 (53.2)	33 (44)	92 (63)	293 (69.7)	60 (71.4)
Physical activity§, ≥4 hr/wk n (%)	22 (19.8)	65 (20.1)	18 (27.7)			
HC user, n (%)	40 (31.2)	113 (30.2)	22 (29.3)	65 (53.3)	226 (62.8)	44 (60.3)
Family Income, n (%)						
≤A$35 000	14 (12.7)	41 (13.1)	10 (15.9)			
A$35 001 to ≤ A$78 000	43 (39.1)	100 (32)	22 (34.9)			
>A$78 000	53 (48.2)	172 (55)	31 (49.2)			

Data are expressed as mean (standard deviation), median (Q1, Q3) †, or n (percentage).

Abbreviations: CVD, cardiovascular disease; BMI, body mass index; SBP, systolic blood pressure; DBP, diastolic blood pressure; HDL-C, high-density lipoprotein cholesterol; LDL-C, low-density lipoprotein cholesterol; HOMA-IR, homeostasis model of assessment for insulin resistance; HC, hormonal contraceptives.

* Smoking ≥ 1 cigarette in a week;

^ǂ^ Consuming alcohol at any level over the last 7 days;

^§^ Having ≥ 4 hr of exercise in free time per week.

There was a linear relationship between age at menarche and current BMI with no evidence that the association varied between 17 and 20 years. Therefore, a single estimate of the effect is reported for both time periods.

After adjusting for the change in the CVD outcome over time and for potential confounders, there was a significant inverse association between age at menarche and BMI at age 17 and 20 years (Table 3). Each later year in age at menarche was associated with a 0.75 kg/m^2^ reduction in BMI (coefficient -0.75 [95%CI -1.06, -0.44]). This association shows increasing BMI with earlier age at menarche. However, this association was no longer statistically significant after further adjustment for BMI at age 8 years (coefficient -0.16 [95%CI -0.42, 0.08]). There was no significant interaction of age at menarche and BMI (expressed as a continuous variable [p = 0.209] (S1 Table) or as tertiles [p = 0.585] (S2 Table)) at age 8 years. There was no significant influence of current smoking, alcohol consumption, physical activity, socio-economic status, or HC use, on the association between age at menarche and BMI at 17 and 20 years. Further, after multiple testing correction, there was no significant association between age at menarche and other CVD risk factors, including SBP, DBP, total cholesterol, HDL-C, LDL-C, triglycerides, glucose, insulin, and HOMA-IR, after adjustment for the change over time in the CVD risk factors (S3 Table).

**Table 3:** Longitudinal hierarchical linear mixed models of the association between age at menarche and BMI at years 17 to 20.

Models	N	Regression coefficient for age at menarche	95% C I	P value
*Included Time* ¶	584	-0.82	-1.16, -0.47	<0.001
*Included Time* ¶ *+ significant Covariates* ǂ *	584	-0.75	-1.06, -0.44	<0.001
*Included Time* ¶ *+ Covariates* ǂ + *BMI at 8 years of age* *	584	-0.16	-0.42, 0.08	0.188

Abbreviations: CI, confidence interval; BMI, body mass index

^¶^ Representing the change in the outcome between ages 17 and 20 years

^ǂ^ Including alcohol consumption and maternal pre-pregnancy BMI

* Variables explored for significant associations include: maternal pre-pregnancy BMI, maternal smoking in pregnancy, birth weight, current smoking, alcohol consumption, hormonal contraceptive use, physical activity, and family income

At 17 and 20 years of age, 19.1% and 21.5%, respectively, were classified as being in a high-risk metabolic cluster, and 4.3% with the metabolic syndrome (Table 1). Characteristics of the high- and low-risk metabolic clusters at 17 and 20 years, and the metabolic syndrome at year 20, are shown in S4 Table. Models for the relationship between age at menarche and the metabolic clusters at years 17 and 20, respectively, and the metabolic syndrome at year 20, are shown in Table 4. For each year of later onset in age at menarche, there was an approximate 30% reduction in the odds of being in the high-risk metabolic cluster at 17 years (OR = 0.73 [95%CI 0.57, 0.94]) and 20 years (OR = 0.68 [95%CI 0.52, 0.87]), and a 40% reduction in the odds of having the metabolic syndrome at year 20 (OR = 0.60 [95% CI 0.41, 0.88]). These data show earlier age at menarche was associated with increased odds of being in the high-risk metabolic cluster or of having the metabolic syndrome. However, these associations were not significant after adjustment for BMI at 8 years. There were no interactions between significant covariates and age at menarche (S5 Table) in the metabolic cluster models at age 17 or 20 years, or the metabolic syndrome at year 20.

**Table 4:** Logistic regression models of the association of age at menarche with the metabolic clusters at years 17 and 20 and the metabolic syndrome at year 20.

	*Metabolic clusters*								*Metabolic syndrome*			
Models	*Year 17*				*Year 20*				*Year 20*			
	N	OR	95% CI	P value	N	OR	95% CI	P value	N	OR	95% CI	P value
*Univariate*	417	0.69	0.54, 0.88	0.003	404	0.62	0.49, 0.80	<0.001	500	0.58	0.40, 0.84	0.004
*Included covariates* ¶ *	417	0.73	0.57, 0.94	0.0015	404	0.68	0.52, 0.87	0.003	500	0.60	0.41, 0.88	0.009
*Included covariates* ¶ + *BMI at 8 years of age* *	417	0.89	0.68, 1.16	0.392	404	0.80	0.61, 1.05	0.114	500	0.73	0.47, 1.11	0.145

Abbreviations: OR, odds ratio; CI, confidence interval; BMI, body mass index

^¶^ Including maternal smoking in pregnancy and maternal pre-pregnancy BMI (y 17), and maternal pre-pregnancy BMI (y 20)

* Variables explored for significant associations include: maternal pre-pregnancy BMI, maternal smoking in pregnancy, birth weight, current smoking, alcohol consumption, hormonal contraceptive use, physical activity, and family income

## Discussion

In a large well-phenotyped pregnancy cohort with prospectively measured age at menarche, we have shown that earlier age at menarche was associated with higher BMI levels at age 17 and 20 years, and increased odds of being in a cluster of high metabolic risk at both ages or of having the metabolic syndrome at 20 years. Importantly, our findings show these associations were largely accounted for by elevated BMI in childhood. There was no significant association between age at menarche and a range of CVD risk factors at 17 and 20 years, including blood pressure, fasting lipids, glucose, insulin and HOMA-IR. Our findings suggest that early age at menarche is not itself an independent risk factor for increased cardio-metabolic risk in late adolescence and young adulthood. Earlier age at menarche (primary school age) may be indicative of a higher risk profile for CVD in young adulthood and hence be a marker for girls requiring earlier intervention (such as weight control) to modify this risk.

Early age at menarche may be viewed as a sentinel event; hence studies of health intervention strategies to mitigate against the subsequent development of cardiometabolic disorders are essential. Studies have shown that the age at menarche has been declining over time, and related to increased adiposity in girls [24]. Over the last two centuries in Europe the mean age at menarche has decreased by 44 days for every five year birth cohort, being greatest in Spain and Germany [25]. Numerous physiological, social and inherited factors are thought to contribute to age at menarche including pre-menarcheal endocrine and nutritional status, and body adiposity [26]. There is extensive evidence that the onset and tempo of puberty are influenced by childhood adiposity [27]. We have previously reported from the Raine cohort that being born with a birthweight below expected by gestation at delivery with adjustment for mother’s height, age and parity, and subsequently having a BMI above average at 8 years of age, significantly predicted early age of menarche [16]. Our current findings suggest that age at menarche is driven by BMI in childhood, and subsequent CVD risk profiles in young adulthood are associated with childhood BMI and not independently with age at menarche.

We focused on BMI related outcomes in this young adulthood period, as a meta-analysis previously reported that the effects of age at menarche on adulthood BMI were greater in younger women [12]. Several studies have reported a significant association between earlier age at menarche and higher adiposity levels in adulthood [8, 12], however only a few assessed the confounding role of childhood BMI [28, 29]. An independent effect of age at menarche on later adiposity was reported in studies with childhood BMI measured at 4 to 6 years [28], but not in others [29], including the present study in which BMI was assessed at 8 years. Our study had the advantage of prospective measures of BMI at 8 years of age, closer to the onset of pubertal maturation and menarche.

The strength of our study has been the use of a large well-characterised contemporary population-based pregnancy cohort, with comprehensive anthropometric, clinical and socio-behavioural data prospectively collected through serial surveys from pregnancy throughout childhood and adolescence to early adulthood. Age at menarche was calculated to the nearest month using a purpose-designed questionnaire at 8, 10, 14 and 17 years of age. Cohort effects have been an important methodological issue in assessing the impact of age at menarche on height [25] or adult cardiovascular disease [30]. By using a single birth cohort, we have excluded cohort bias. Furthermore, we employed hierarchical mixed effects statistical analysis that enabled a longitudinal assessment of the development of CVD outcomes over time, in models that considered the confounding effects of a range of covariates and their interaction with time. Our population was predominantly Caucasian, and was largely of middle to upper level socio economic status (SES). This limits the ability of our study to comment on how ethnicity may impact on the relationship between age at menarche and CVD risk, but also reduces important sources of bias since both ethnicity and SES are known to influence age at menarche [31].

In summary, our study shows that age at menarche is not an independent predictor for cardiometabolic risk in late adolescence and young adulthood. Our findings suggest that the association between early menarche and cardiometabolic risk factors is due to underlying childhood adiposity, suggesting that targeting childhood adiposity may increase both age at menarche and CV health in later life. For girls who experience menarche at a younger (primary school) age who have modifiable risk factors for CVD (such as obesity) targeted interventions such as weight reduction may reduce the risk of CVD in later life.

## Supporting information

S1 Table Linear mixed model of adulthood BMI: Interaction between age at menarche and BMI at age 8 years. (DOCX)

S2 Table Linear mixed model of adulthood BMI: Interaction of tertiles of BMI at age 8 years with age at menarche. (DOCX)

S3 Table Longitudinal mixed model regression associations between age at menarche and cardiovascular risk factors. (DOCX)

S4 Table Components of the metabolic clusters at 17 and 20 years and the metabolic syndrome at 20 years. (DOCX)

S5 Table Interactions of age at menarche with significant covariates in models for the metabolic clusters at years 17 and 20, and the metabolic syndrome at year 20. (DOCX)
