# Supplementary material for: Age at menarche and childhood body mass index as predictors of cardio-metabolic risk in young adulthood: A prospective cohort study
Source: PLoS One. 2018 Dec 21;13(12):e0209355. doi: 10.1371/journal.pone.0209355 (PMC6303033; doi:10.1371/journal.pone.0209355)
Supplement: S1 Table — (DOCX) [file pone.0209355.s001.docx]

**S1 Table. Linear mixed model of adulthood BMI: Interaction between age at menarche and BMI at age 8 years**

|  | **Regression coefficient** | **95% CI** | **P value** |
| --- | --- | --- | --- |
| Year | 0.31 | 0.26, 0.37 | <0.001 |
| Age at menarche | -0.14 | -0.42, 0.14 | 0.334 |
| BMI at age 8 years | 1.28 | 1.15, 1.40 | <0.001 |
| Interaction of BMI at age 8 years with age at menarche | 0.06 | -0.03, 0.16 | 0.209 |
| *Constant* | *18.16* | *17.08, 19.23* | *<0.001* |
