# Supplementary material for: Age at menarche and childhood body mass index as predictors of cardio-metabolic risk in young adulthood: A prospective cohort study
Source: PLoS One. 2018 Dec 21;13(12):e0209355. doi: 10.1371/journal.pone.0209355 (PMC6303033; doi:10.1371/journal.pone.0209355)
Supplement: S2 Table — (DOCX) [file pone.0209355.s002.docx]

**S2 Table. Linear mixed model of adulthood BMI: Interaction of tertiles of BMI at age 8 years with age at menarche**

|  | **Regression coefficient** | **95% CI** | **P value** |
| --- | --- | --- | --- |
| Year | 0.31 | 0.25, 0.37 | <0.001 |
| Age at menarche | -0.53 | -1.11, 0.04 | 0.067 |
| Tertiles of BMI at age 8 years |  |  |  |
| 2 | 1.84 | 1.01, 2.68 | <0.001 |
| 3 | 5.87 | 5.03, 6.72 | <0.001 |
| Interaction of BMI at age 8 years with age at menarche |  |  | 0.585 |
| 2 | 0.35 | -0.44, 1.14 | 0.391 |
| 3 | -0.01 | -0.78, 0.76 | 0.98 |
| *Constant* | *15.61* | *14.42, 16.81* | *<0.001* |
