# Supplementary material for: Age at menarche and childhood body mass index as predictors of cardio-metabolic risk in young adulthood: A prospective cohort study
Source: PLoS One. 2018 Dec 21;13(12):e0209355. doi: 10.1371/journal.pone.0209355 (PMC6303033; doi:10.1371/journal.pone.0209355)
Supplement: S3 Table — (DOCX) [file pone.0209355.s003.docx]

**S3 Table Longitudinal mixed model regression associations between age at menarche and cardiovascular risk factors**

|  | **Regression coefficient**  **for age at menarche** | **95% CI** | **P** |
| --- | --- | --- | --- |
| SBP † | -0.54 | -1.14, 0.06 | 0.076 |
| DBP † | -0.44 | -0.87, 0.01 | 0.045 |
| Total cholesterol ǂ | -0.04 | -0.09, 0.01 | 0.147 |
| Triglycerides ǂ | -0.002 | -0.03, 0.03 | 0.921 |
| HDL-C ǂ | -0.008 | -0.03, 0.01 | 0.486 |
| LDL-C ǂ | -0.03 | -0.07, 0.01 | 0.151 |
| Glucose ǂ | -0.006 | -0.05, 0.03 | 0.742 |
| Insulin ǂ | -0.31 | -0.73, 0.11 | 0.146 |
| HOMA-IR ǂ | -0.06 | -0.16, 0.03 | 0.183 |

† N = 650; ǂ N = 569

Hierarchical linear mixed models of age at menarche with CVD risk factors measured at years 17 and 20, adjusted for the change over time between 17 and 20 years.

Abbreviations: CI, confidence interval; BMI, body mass index; SBP, systolic blood pressure; DBP, diastolic blood pressure; HDL-C, high-density lipoprotein cholesterol; LDL-C, low-density lipoprotein cholesterol; HOMA-IR, homeostasis model of assessment for insulin resistance.
