# Supplementary material for: Age at menarche and childhood body mass index as predictors of cardio-metabolic risk in young adulthood: A prospective cohort study
Source: PLoS One. 2018 Dec 21;13(12):e0209355. doi: 10.1371/journal.pone.0209355 (PMC6303033; doi:10.1371/journal.pone.0209355)
Supplement: S4 Table — (DOCX) [file pone.0209355.s004.docx]

**S4 Table Components of the metabolic clusters at 17 and 20 years of age, and the metabolic syndrome at 20 years**

|  | ***Metabolic clusters*** | | | | ***Metabolic syndrome*** | |
| --- | --- | --- | --- | --- | --- | --- |
|  | **Year 17** | | **Year 20** | | **Year 20** | |
|  | ***High risk*** *(n=87)* | ***Low risk*** *(n=368)* | ***High risk*** *(n=96)* | ***Low risk*** *(n=350)* | ***With***  *(n=24)* | ***Without***  *(n=527)* |
| Current BMI, kg/m^2^ | 29.8 (5.2) | 21.7 (2.5) | 31.5 (6.2) | 22.3 (2.6) |  |  |
| Waist circumference, cm |  |  |  |  | 101.3 (14.8) | 75.9 (11.1) |
| SBP, mmHg | 114.9 (10.9) | 107.5 (8.1) | 118.2 (10.3) | 108.9 (8.8) | 122.01 (12.3) | 110.57 (9.5) |
| DBP, mmHg |  |  |  |  | 71.95 (9.2) | 65.05 (6.9) |
| Serum triglycerides, mmol/L | 1.44 (0.8) | 0.94 (0.3) | 1.39 (0.5) | 0.97 (0.4) | 1.80 (0.6) | 1.01 (0.4) |
| Serum HDL-C, mmol/L |  |  |  |  | 1.07 (0.2) | 1.47 (0.3) |
| Glucose, mmol/L |  |  |  |  | 5.16 (0.4) | 4.85 (0.7) |
| HOMA-IR † | 2.85 (2.1, 4.4) | 1.45 (1, 2.1) | 1.77 (1.2, 2.5) | 0.46 (0.4, 0.8) |  |  |

Data are expressed as mean (standard deviation), or median (Q1, Q3) †

Abbreviations: BMI, body mass index; SBP, systolic blood pressure; HDL-C, high-density lipoprotein cholesterol; HOMA-IR, homeostasis model of assessment for insulin resistance
