# Supplementary material for: Age at menarche and childhood body mass index as predictors of cardio-metabolic risk in young adulthood: A prospective cohort study
Source: PLoS One. 2018 Dec 21;13(12):e0209355. doi: 10.1371/journal.pone.0209355 (PMC6303033; doi:10.1371/journal.pone.0209355)
Supplement: S5 Table — (DOCX) [file pone.0209355.s005.docx]

**S5 Table. Interactions of age at menarche with significant covariates in models for the metabolic clusters at years 17 and 20, and the**

**metabolic syndrome at year 20**

|  | ***Metabolic clusters*** | | | | | | ***Metabolic syndrome*** | | |
| --- | --- | --- | --- | --- | --- | --- | --- | --- | --- |
| **Interaction of age at menarche with** | ***Year 17***  *N=417* | | | ***Year 20***  *N=404* | | | ***Year 20***  *N=500* | | |
|  | **OR** | **95% CI** | **Interaction p value** | **OR** | **95% CI** | **Interaction p value** | **OR** | **95% CI** | **Interaction p value** |
| *BMI at age 8 years* | 1.07 | 0.96, 1.19 | 0.20 | 0.98 | 0.86, 1.12 | 0.79 | 1.04 | 0.91, 1.2 | 0.59 |
| *Maternal prenatal BMI* | 1.01 | 0.96, 1.05 | 0.72 | 1.00 | 0.94, 1.06 | 0.94 | 0.93 | 0.86, 1.01 | 0.09 |
| *Maternal smoking in pregnancy* | 0.76 | 0.41, 1.4 | 0.38 |  |  |  |  |  |  |
